# Supplementary material for: Non-Linear Frequency Dependence of Neurovascular Coupling in the Cerebellar Cortex Implies Vasodilation–Vasoconstriction Competition
Source: Cells. 2022 Mar 19;11(6):1047. doi: 10.3390/cells11061047 (PMC8947624; doi:10.3390/cells11061047)
Supplement: Supplementary file 1 [file cells-11-01047-s001.zip › cells-1610364-supplementary.pdf]

cells

MDPI

Original Article

Non-linear frequency-dependence of neurovascular coupling in the cerebellar cortex implies vasodilation-vasoconstriction competition

Giuseppe Gagliano[1*], Anita Monteverdi[1,3*], Stefano Casali[1], Umberto Laforenza[2], Claudia A.M. Gandini Wheeler-Kingshott[4,1,3], Egidio D'Angelo[1,3#], Lisa Mapelli[1#]

[1] Department of Brain and Behavioral Sciences, University of Pavia, Pavia, Italy
[2] Department of Molecular Medicine, University of Pavia, Pavia, Italy
[2] IRCCS Mondino Foundation, Pavia, Italy[4] NMR Research Unit, Department of Neuroinflammation, Queen Square MS Centre, UCL Queen Square Institute of Neurology, Faculty of Brain Sciences, University College London, London, United Kingdom

* Co-first authors
\# Co-last and corresponding authors (dangelo@unipv.it; lisa.mapelli@unipv.it)

Supplementary Materials:

Supplementary Materials and Methods

Preparation of acute cerebellar slices

Briefly, mice were anesthetized with halothane (Aldrich, Milwaukee, WI) and killed by decapitation. Acute parasagittal slices of 220μm thickness were cut from the cerebellar vermis and hemisphere with a vibroslicer (VT1200S, Leica Microsystems). During the cutting procedure, slices were maintained in cold Krebs solution. Then, slices were recovered for at least 1h in the same solution before being incubated for 1h with 75nM U46619 (Abcam), a thromboxane agonist. This procedure is commonly used to restore the original vascular tone in acute slices [1-4]. For recording, slices were transferred to a 2-ml recording chamber mounted on the stage of an upright microscope (Slicescope, Scientifica Ltd, UK) or to the recording chamber of the HD-MEA (Arena biochip, Biocam X, 3Brain AG). In both cases, Krebs solution was perfused (2 ml/min) and maintained at 37°C with a Peltier feedback device (TC-324B, Warner Instruments, Hamden, CT) for the entire duration of the experiments. Krebs solution for slice cutting and recovery contained (in mM): 120 NaCl, 2 KCl, 1.2 $MgSO_4$, 26 $NaHCO_3$, 1.2 KH_2PO_4, 2 $CaCl_2$, and 11 glucose, and was equilibrated with 95% O_2-5% CO_2 (pH 7.4). Krebs solution perfused during recordings was added of 75nM U46619. All drugs were obtained from Sigma Aldrich, unless otherwise specified.

Immunofluorescence staining

First, slices (220 μm-thick) were fixed with 4% paraformaldehyde in PBS for 25 min in a Petri dish. Secondly, slices were washed three times before being permeabilized and blocked with 5% Triton X-100, 10% BSA added to PBS, overnight at 4°C. Thirdly, samples were incubated for 24 h at 25°C on a rotary shaker with rabbit anti-NG2 chondroitin sulfate proteoglycan (Millipore, cat no. AB5320, 1:200 dilution) and FITC-isolectin B4 (Sigma-Aldrich, cat. no. L2895; 1:200 dilution of a stock solution of 2 mg/ml FITC-IB4 in PBS) primary antibodies to stain respectively pericytes and blood vessels. Before the incubation with secondary antibodies, slices were washed three times (each of 15 min) with PBS. Fourthly, samples were incubated for 4 h at 25°C with fluorescent secondary antibody Rhodamine Red-X-AffiniPure Goat Anti-Rabbit IgG (Jackson ImmunoResearch Inc. cat. no.111-295-045; 1:500 dilution). Finally, slices were washed three times with PBS and

mounted on miscroscope slides, ProLong® Gold antifade reagent with DAPI (Molecular Probes) and coverslips affixed. Fluorescence of cerebellar samples was observed with a TCS SP5 II LEICA confocal microscopy system (Leica Microsystems, Italy) furnished with a LEICA DM IRBE inverted microscope. The confocal system exploited a 20X, 40X, and 63X objectives to acquire images. All acquisition files were visualized by LAS AF Lite software (Leica Application Suite Advanced Fluorescence Lite version 2.6.0) installed on a desktop PC. Negative controls were carried out in parallel by treating slices with non-immune serum during the incubation procedures.

Estimates of changes in cerebrovascular parameters

The cerebrovascular flow is CBF=CPP/CVR (cerebral perfusion pressure and cerebrovascular resistance), where, for the Poiseuille law: $CVR = 8\ \eta l\ /\ \pi r^4$ (η is viscosity, l is the length of vessel, r is vessel radius). The capillary bed is reported to provide 45% of the resistance in the precapillary districts [5]. Therefore, our calculations will account for this factor that rescales the radius r. Accordingly, a 2.5-7.5% dilation would decrease CVR by 4.5-14.2%. Since CBF is inversely proportional to CVR, CBF will increase by 4.8-16.5% (e.g., 100% / (100%-4.8%)[5, 6]). The cerebral blood volume, CBV, is proportional to r^2 and CBF to r4, then CBV is proportional to CBF0.5. CBV will thus increase by 2.4-7.9% (see 6 and supplementary in 5 for details).\ These estimates are in good agreement with the changes in CBV (ranging from 4 to 10%) measured during the acquisition of BOLD responses in rodents in vivo [7, 8].

Estimate of the number of neurons contributing to the LFPs in HD-MEA recordings

Considerations can be made to obtain an estimate of the number of granule cells/electrode contributing to the LFP response, given the anatomy of the layer and the specifics of the sensors. The main features to take into account are that: i) granule cells are densely packed in the layer; ii) the electrode is recessed by about 1.5μm in the insulating layer of the chip, so the sensitivity is mostly restricted to the neurons covering the electrode itself (with negligible contribution of nearby neurons); iii) the average diameter of granule cells soma is about 5μm [9] (confirmed by our histology, cfr.Fig.1A). Therefore, an electrode of 21x21 μm^2 will be covered by approximately 16 cells. It is reasonable to consider that the electrode will sense neurons activity from the upper layer of cells, bringing the total number to 32 cells. However, not all granule cells are recruited during the stimulation in our condition, reasonably less than 50% (comparing the level of stimulation intensities with previous works in the same lab addressing mossy fiber recruitment at increasing stimulation intensities, e.g., [10]). The estimate of active neurons contributing to the signal recorded in a single electrode is therefore an average of 15. It is reasonable then to specify a range of 10-15 cells per electrode, though it might be underestimated.

Stimulation patterns

Mossy fibers were stimulated at different frequencies in a range from 6Hz to 300Hz, thus spanning the main frequency bands which are known to characterize brain dynamics [11]. In particular, the specific frequencies used have been associated to cerebellar activity. The theta band (6Hz) in the granular layer is reported to characterize cerebellar activity during sensorimotor integration and certain cognitive states [12]. Moreover, the cerebellar cortex shows oscillation and resonance peaking in the theta band, which affects plasticity both *ex vivo* and *in vivo* [13-15]. The beta band (20Hz) is known to characterize cerebellar cortex activity (primarily the granular layer), together with motor cortex one, during voluntary movements and behavior as active and passive expectancy in primates [16-19]. Cerebellar huge interconnectivity with the pontine nuclei is fundamental for sensorimotor functions [20, 21]. These connections are known to operate in the gamma band (low 50Hz and fast 100Hz) where these ranges of activity induce synaptic facilitation in rat pontine nuclei neurons [22, 23]. Finally, the mossy fiber-granule cell connection has been reported to work

in the ultra-fast band (300Hz) *in vivo*, ensuring high-fidelity transmission[24-26]. This was reported as instantaneous frequencies during brief burst, while average mossy fiber discharge is usually below 200Hz. See Discussion for an extended comment.

Computational model of the granular layer

Simulations have been performed using a detailed computational model which reproduces the anatomo-functional organization of the cerebellar granular layer (GL) network [27, 28] endowed with biophysically realistic single cell models of granule cells (GrCs) and Golgi cells (GoCs), as well as a simplified reconstruction of mossy fibers terminals: the glomeruli (gloms). Mossy fibers branch inside the granular layer and terminate in the glomeruli, characterized by an external glial ensheathment, and form synapses with granule cells in a radius of 30µm [29]. Stimulating the mossy fiber bundle determines the activation of the mossy fiber terminals located inside the glomeruli. As for the way it is constructed, the model does not need to stimulate the mossy fibers but, equivalently, directly activates the corresponding glomeruli. Detailed reconstruction of synaptic dynamics and receptors was also included, allowing to reproduce the effect of α-amino-3-hydroxy-5-methyl-4-isoxazolepropionic acid (AMPA), NMDA, and gamma-aminobutyric acid (GABA) receptors mediated currents. The full GL network had a volume of 800 x 800 x 150 μm^3; circuit organization and connectivity has been reproduced according to specific connectivity rules, accounting for both geometric and statistical data (see also [30] for a similar approach). The original version of the granular layer network faithfully reproduced the cell-density distributions experimentally observed in the cerebellar vermis: the full network included 384000 GrCs, 914 GoCs and 29415 gloms. In order to reconstruct a network of the cerebellar hemisphere, different density distributions had been considered: cell data coming from the Allen Brain Atlas (mouse data, https://mouse.brain-map.org/) for the Crus (i.e., the hemisphere) have been collected, analyzed, and compared to those coming from the Lingula (i.e., the vermis). A comparative analysis showed that GrCs and GoCs densities in the hemisphere were respectively 64% and 89% higher than in the vermis. Finally, gloms density has been estimated assuming that the GrCs / gloms ratio should be kept constant. This led to a network reconstruction of the hemisphere-GL with 630419 GrCs, 1.733 GoCs and 48291 gloms. It should also be noted that the GrCs / GoCs ratio changed significantly from the vermis (~ 420:1) to the hemisphere (~ 364:1).

The experimental protocol has been reproduced with the network model as follows: a subset of gloms was stimulated with continuous trains at five different frequencies, 6, 20, 50, 100, and 300 Hz, 35 seconds per simulation. Gloms were selected according to a simple geometrical rule (see also [27]): all the gloms included within a sphere centered in the middle of the GL network and with radius equal to 27.7µm were stimulated with the same train. Given the different cell-densities, the total amount of excited GrCs was 1290 and 1996 for vermis and hemisphere networks, respectively. Notice that the number of GrCs contributing to the simulated response is comparable to the estimated total number of GrCs contributing to the LFP signal (see above: 10-15 neurons per electrode, 9 electrodes per slice, 10 slices per condition, resulting in several neurons contributing to the total average LFP above 900-1350 units).

NMDAR-mediated currents were recorded for each stimulated GrC. In order to compare NO production to NMDA currents over time, the cumulative sum of NMDA-current, averaged over each cell, was calculated at different time points: 2, 20, and 35 seconds of the stimulation period. The computational model of GL network has been developed in the NEURON-Python simulation environment; simulations were performed on a 60 cores local cluster (five blades with two Intel Xeon X560 and 24 Gigabyte of DDR3 ram per blade, twelve cores per blade).

Supplementary figure

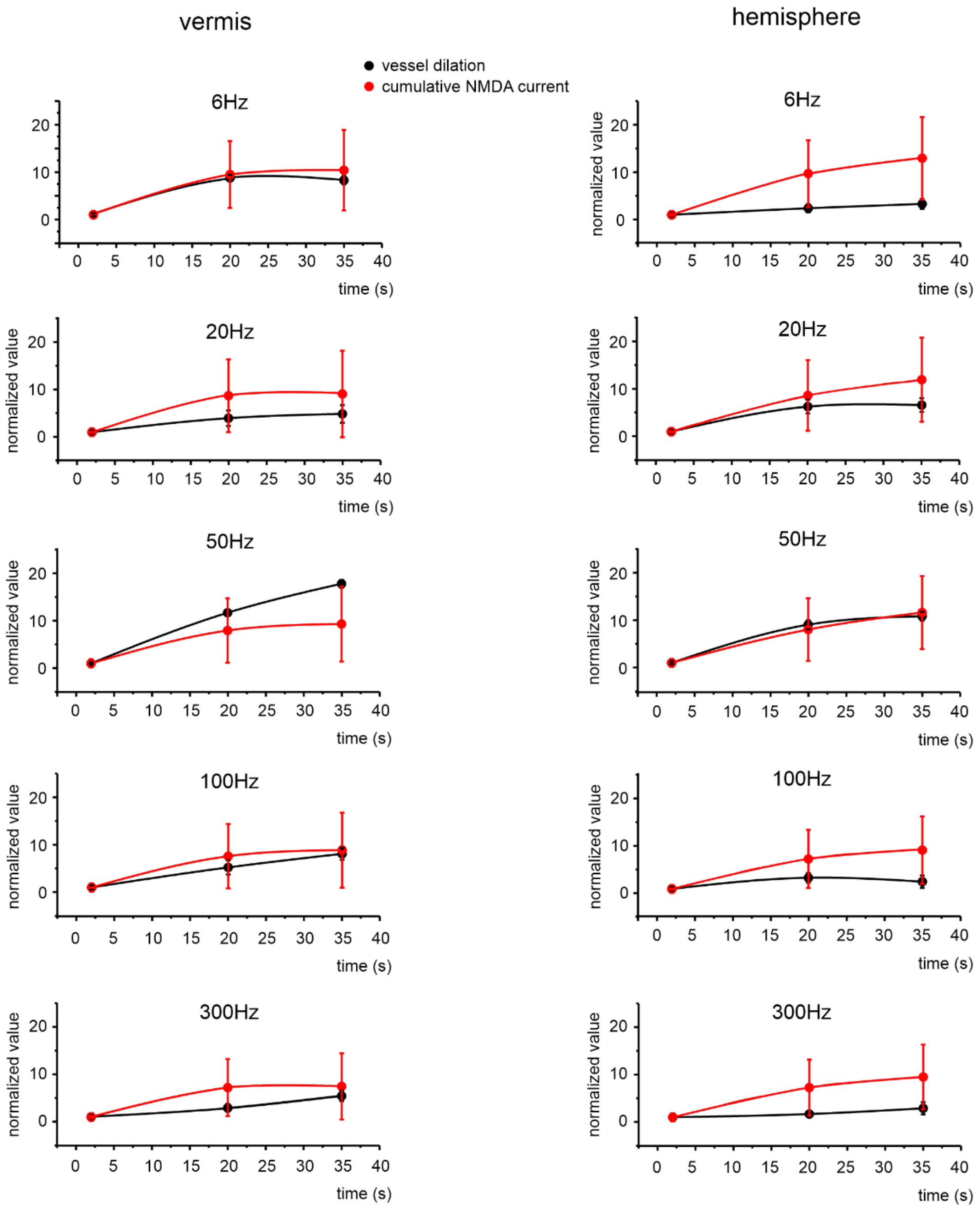

Figure S1. – Model-derived NMDA component of granule cell responses: comparison to vessel response at different stimulation frequencies. Time-course of the granule cells cumulative NMDA current (*red*) building-up during the simulation at different frequencies and of the vessel responses (*black*) in the corresponding conditions. Both parameters are normalized for their amplitude at 2s, to compare the trends over the stimulation time. Notice that the trends of the two parameters do not significantly differ at any frequency tested, both for the vermis (*at left*) and hemisphere (*at right*).

Table S1. – Maximal capillary dilations at different stimulation frequencies. The table reports the average maximal dilations (percent changes compared to the baseline) observed at different stimulation frequencies in the vermis and hemisphere (paired Student's t test: *p<0.05; **p<0.01; ***p<0.001).

	6Hz	20Hz	50Hz	100Hz	300Hz
**vermis**	2.99±0.61(***)	7.19±1.85(***)	3.66±0.82(***)	7.24±1.26(***)	6.42±1.10(***)
**hemisphere**	4.93±1.06(**)	6.96±1.45(***)	5.79±0.91(***)	4.28±1.00(***)	7.04±1.28(***)

Table S2. – N_{2a} and N_{2b} peak amplitude changes at the end of the stimulations. The table reports the average percent changes of N_{2a} and N_{2b} peak amplitudes comparing the last response to the first one in the stimulation train, at the different frequencies used (paired Student's t test: *p<0.05; **p<0.01; ***p<0.001).

	6Hz	20Hz	50Hz	100Hz
**vermis N2a**	-20.3 ± 4.3 (*)	-41.7 ± 4.4 (***)	-52.4 ± 3.9 (***)	-52.2 ± 3.4 (***)
**vermis N2b**	-3.0 ± 4.3 (**)	-12.3 ± 4.5 (***)	-7.2 ±5.6 (***)	-2.8 ± 6.3 (***)
**hemisphere N2a**	-22.1 ± 4.4 (***)	-47.9 ± 3.9 (***)	-54.9 ± 2.5 (***)	-54.7 ± 2.8 (***)
**hemisphere N2b**	-23.1 ± 6.4 (***)	-22.8 ± 6.2 (***)	-29.1 ± 5.5 (***)	-23.0 ± 5.5 (***)

Supplementary Materials Reference list

1. Rancillac A, Rossier J, Guille M, et al. Glutamatergic Control of Microvascular Tone by Distinct GABA Neurons in the Cerebellum. J Neurosci 2006; 26: 6997-7006. 2006/06/30. DOI: 10.1523/jneurosci.5515-05.2006.
2. Fernandez-Klett F, Offenhauser N, Dirnagl U, et al. Pericytes in capillaries are contractile in vivo, but arterioles mediate functional hyperemia in the mouse brain. Proc Natl Acad Sci U S A 2010; 107: 22290-22295. 2010/12/08. DOI: 10.1073/pnas.1011321108.
3. Mishra A, O'Farrell FM, Reynell C, et al. Imaging pericytes and capillary diameter in brain slices and isolated retinae. Nat Protoc 2014; 9: 323-336. 2014/01/18. DOI: 10.1038/nprot.2014.019.
4. Mapelli L, Gagliano G, Soda T, et al. Granular Layer Neurons Control Cerebellar Neurovascular Coupling Through an NMDA Receptor/NO-Dependent System. J Neurosci 2017; 37: 1340-1351. 2016/12/30. DOI: 10.1523/JNEUROSCI.2025-16.2016.
5. Peppiatt CM, Howarth C, Mobbs P, et al. Bidirectional control of CNS capillary diameter by pericytes. Nature 2006; 443. DOI: 10.1038/nature05193.
6. Bogorad M, DeStefano J, Linville R, et al. Cerebrovascular plasticity: Processes that lead to changes in the architecture of brain microvessels. J Cereb Blood Flow Metab 2019; 39. DOI: 10.1177/0271678X19855875.
7. Huber L, Poser B, Kaas A, et al. Validating layer-specific VASO across species. NeuroImage 2021; 237. DOI: 10.1016/j.neuroimage.2021.118195.
8. Lu H, Patel S, Luo F, et al. Spatial correlations of laminar BOLD and CBV responses to rat whisker stimulation with neuronal activity localized by Fos expression. Magn Reson Med 2004; 52. DOI: 10.1002/mrm.20265.
9. D'Angelo E. Cerebellar Granule Cell. In: Manto M, Schmahmann JD, Rossi F, et al. (eds) Handbook of the Cerebellum and Cerebellar Disorders. Dordrecht: Springer, 2013.
10. Sola E, Prestori F, Rossi P, et al. Increased neurotransmitter release during long-term potentiation at mossy fibre-granule cell synapses in rat cerebellum. J Physiol 2004; 557: 843-861. DOI: 10.1113/jphysiol.2003.060285.
11. Buzsáki G. Rhythms of the Brain. New York, US: Oxford University Press, 2006.
12. De Zeeuw CI, Hoebeek FE and Schonewille M. Causes and Consequences of Oscillations in the Cerebellar Cortex. Neuron 2008; 58: 655-658. DOI: https://doi.org/10.1016/j.neuron.2008.05.019.
13. D'Angelo E, Solinas S, Mapelli J, et al. The cerebellar Golgi cell and spatiotemporal organization of granular layer activity. Front Neural Circuits 2013; 7: 93. DOI: 10.3389/fncir.2013.00093.
14. Sgritta M, Locatelli F, Soda T, et al. Hebbian Spike-Timing Dependent Plasticity at the Cerebellar Input Stage. J Neurosci 2017; 37: 2809-2823. 2017/02/12. DOI: 10.1523/jneurosci.2079-16.2016.
15. Moscato L, Montagna I, De Propris L, et al. Long-Lasting Response Changes in Deep Cerebellar Nuclei. Front Cell Neurosci 2019; 13: 84. 2019/03/06. DOI: 10.3389/fncel.2019.00084.
16. Pellerin JP and Lamarre Y. Local field potential oscillations in primate cerebellar cortex during voluntary movement. J Neurophysiol 1997; 78: 3502-3507. 1998/02/07. DOI: 10.1152/jn.1997.78.6.3502.
17. Donoghue JP, Sanes JN, Hatsopoulos NG, et al. Neural discharge and local field potential oscillations in primate motor cortex during voluntary movements. J Neurophysiol 1998; 79: 159-173. 1998/02/21. DOI: 10.1152/jn.1998.79.1.159.
18. Courtemanche R, Pellerin JP and Lamarre Y. Local field potential oscillations in primate cerebellar cortex: modulation during active and passive expectancy. J Neurophysiol 2002; 88: 771-782. 2002/08/07. DOI: 10.1152/jn.2002.88.2.771.
19. Courtemanche R and Lamarre Y. Local field potential oscillations in primate cerebellar cortex: synchronization with cerebral cortex during active and passive expectancy. J Neurophysiol 2005; 93: 2039-2052. 2004/12/14. DOI: 10.1152/jn.00080.2004.
20. Bjaalie JG and Leergaard TB. Functions of the pontine nuclei in cerebro-cerebellar communication. Trends Neurosci. England, 2000, pp.152-153.
21. Nagao S. Pontine nuclei-mediated cerebello-cerebral interactions and its functional role. Cerebellum 2004; 3: 11-15. 2004/04/10. DOI: 10.1080/14734220310012181.
22. Mock M, Schwarz C and Thier P. Electrophysiological properties of rat pontine nuclei neurons In vitro II. Postsynaptic potentials. J Neurophysiol 1997; 78: 3338-3350. 1998/02/07. DOI: 10.1152/jn.1997.78.6.3338.
23. Schwarz C, Mock M and Thier P. Electrophysiological properties of rat pontine nuclei neurons In vitro. I. Membrane potentials and firing patterns. J Neurophysiol 1997; 78: 3323-3337. 1998/02/07. DOI: 10.1152/jn.1997.78.6.3323.

24. Rancz EA, Ishikawa T, Duguid I, et al. High-fidelity transmission of sensory information by single cerebellar mossy fibre boutons. Nature 2007; 450: 1245-1248. 2007/12/22. DOI: 10.1038/nature05995.
25. van Beugen BJ, Gao Z, Boele HJ, et al. High frequency burst firing of granule cells ensures transmission at the parallel fiber to purkinje cell synapse at the cost of temporal coding. Front Neural Circuits 2013; 7: 95. 2013/06/05. DOI: 10.3389/fncir.2013.00095.
26. Delvendahl I and Hallermann S. The Cerebellar Mossy Fiber Synapse as a Model for High-Frequency Transmission in the Mammalian CNS. Trends Neurosci 2016; 39: 722-737. 2016/10/25. DOI: 10.1016/j.tins.2016.09.006.
27. Casali S, Tognolina M, Gandolfi D, et al. Cellular-resolution mapping uncovers spatial adaptive filtering at the rat cerebellum input stage. Commun Biol 2020; 3. DOI: 10.1038/s42003-020-01360-y.
28. De Schepper R, Geminiani A, Masoli S, et al. Scaffold modelling captures the structure-function-dynamics relationship in brain microcircuits. boRxiv 2021. DOI: 10.1101/2021.07.30.454314.
29. Palay SL and Chan-Palay V. Cerebellar Cortex: Cytology and Organization. New York: Springer-Verlag, 1974.
30. Solinas S, Nieus T and D'Angelo E. A realistic large-scale model of the cerebellum granular layer predicts circuit spatio-temporal filtering properties. Front Cell Neurosci 2010; 4: 12. DOI: 10.3389/fncel.2010.00012.
